# Safety and efficacy of intra-arterial tenecteplase for non-complete reperfusion of intracranial occlusions: Methodology of a randomized, controlled, multicenter study

**DOI:** 10.1093/esj/23969873251381974

**Published:** 2026-01-01

**Authors:** Johannes Kaesmacher, Adnan Mujanovic, Seraina Beyeler, Lukas Bütikofer, Morin Beyeler, Eike I Piechowiak, Tomas Dobrocky, Mira Katan, Pasquale Mordasini, Grégoire Boulouis, Zsolt Kulcsar, Marios Psychogios, Mikael Mazighi, Daniel Strbian, Götz Thomalla, Robin Lemmens, Jan-Hendrik Schäfer, Franziska Dorn, Olav Jansen, Roland Schwab, Helge Kniep, Jens Fiehler, Wenjie Zi, Jan Gralla, Urs Fischer

**Affiliations:** Department of Diagnostic and Interventional Neuroradiology, University Hospital Bern Inselspital, University of Bern, Bern, Switzerland; Clinical Investigation Center of Tours – Technological Innovation and Department of Neuroradiology, CHU Tours, Tours, France; Department of Diagnostic and Interventional Neuroradiology, University Hospital Bern Inselspital, University of Bern, Bern, Switzerland; Department of Diagnostic and Interventional Neuroradiology, University Hospital Bern Inselspital, University of Bern, Bern, Switzerland; Department of Neurology, University Hospital Bern Inselspital, University of Bern, Bern, Switzerland; Department of Clinical Research, CTU Bern, University of Bern, Bern, Switzerland; Department of Neurology, University Hospital Bern Inselspital, University of Bern, Bern, Switzerland; Department of Diagnostic and Interventional Neuroradiology, University Hospital Bern Inselspital, University of Bern, Bern, Switzerland; Department of Diagnostic and Interventional Neuroradiology, University Hospital Bern Inselspital, University of Bern, Bern, Switzerland; Department of Neurology, University Hospital Basel, University of Basel, Basel, Switzerland; Institute of Diagnostic and Interventional Neuroradiology, Center for Imaging and Minimally Invasive Therapies, Kantonsspital Aarau (KSA), Aarau, Switzerland; Clinical Investigation Center of Tours – Technological Innovation and Department of Neuroradiology, CHU Tours, Tours, France; Department of Neuroradiology, Clinical Neuroscience Center, University Hospital Zurich, Zurich, Switzerland; Department of Neuroradiology, University Hospital Basel, University of Basel, Basel, Switzerland; Department of Neurology, Hôpital Lariboisière, FHU Neurovasc, Université Paris Cité, Inserm, Paris, France; Department of Neurology, Helsinki University Hospital, University of Helsinki, Helsinki, Finland; Department of Neurology, University Medical Center Hamburg-Eppendorf, Hamburg, Germany; Department of Neurology, University Hospital Leuven, Leuven Brain Institute, Leuven, Belgium; Department of Neurology, Goethe University Frankfurt, University Hospital, Frankfurt, Germany; Department of Neuroradiology, University Hospital Bonn, Bonn, Germany; Department of Radiology and Neuroradiology, University Hospital Kiel, Kiel, Germany; University Clinic for Neuroradiology, University Hospital Magdeburg, Magdeburg, Germany; Department of Diagnostic and Interventional Neuroradiology, Hamburg University, Hamburg, Germany; Department of Diagnostic and Interventional Neuroradiology, Hamburg University, Hamburg, Germany; Department of Neurology, Xinqiao Hospital and the Second Affiliated Hospital, Army Medical University (Third Military Medical University), Chongqing, China; Department of Diagnostic and Interventional Neuroradiology, University Hospital Bern Inselspital, University of Bern, Bern, Switzerland; Department of Neurology, University Hospital Bern Inselspital, University of Bern, Bern, Switzerland

**Keywords:** Reperfusion, tenecteplase, intra-arterial, TICI

## Abstract

**Rationale:**

Intra-arterial fibrinolytics may be used for distal remaining vessel occlusions after incomplete mechanical thrombectomy (MT). However, their efficacy in improving reperfusion in this specific clinical scenario is unclear. While better reperfusion may lead to improved clinical outcomes, additional fibrinolytics could also increase the risk of hemorrhagic complications.

**Aim:**

To assess the safety and reperfusion efficacy of intra-arterial tenecteplase (TNK) compared to no further interventional treatment in patients with incomplete reperfusion and mechanically non-amendable residual occlusions after MT.

**Methods and design:**

This is an international, multicenter, randomized (1:1) controlled, two-arm, open, assessor-blinded, surrogate endpoint trial. The interventional arm receives 3 mg (not weight-adjusted) intra-arterial TNK, administered as close as possible to the residual occlusion. The control arm receives no further interventional treatment.

**Sample size:**

TECNO will randomize 156 participants 1:1 to 3 mg intra-arterial tenecteplase or no further interventional treatment. This sample size is based on anticipated absolute improvements in early and late reperfusion with intra-arterial TNK of 25% and 30%, respectively.

**Outcomes:**

The two co-primary imaging outcomes are early and late reperfusion. Early reperfusion is defined as an extended Thrombolysis in Cerebral Infarction (eTICI) score ⩾ 2a for residual occlusions on angiography 25 min after randomization. Late reperfusion is defined as the absence of a wedge-shaped perfusion delay on delay-sensitive perfusion maps assessed on 24 h ± 6 h perfusion imaging. Standard secondary clinical outcomes will be assessed at 24 h and 90 ± 15 days.

**Discussion:**

The TECNO trial will provide evidence on the safety and reperfusion efficacy of locally administered intra-arterial TNK in patients with residual occlusions following MT.

## Graphical Abstract

**Graphical Abstract:**
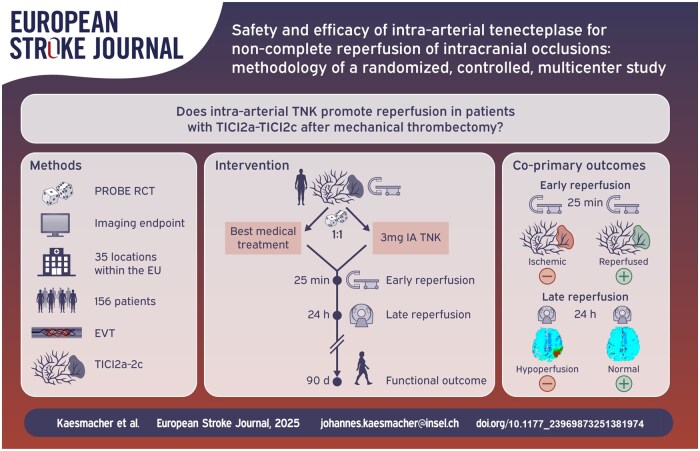

## Introduction

Timely and complete reperfusion is a key determinant of favorable outcomes and mortality for patients with acute ischemic stroke undergoing mechanical thrombectomy (MT).^1,2^ Hence, current European Stroke Organization (ESO) and European Society for Minimally Invasive Neurological Therapy (ESMINT) guidelines recommend striving for near-complete or complete reperfusion (defined as extended Thrombolysis in Cerebral Infarction score, eTICI 2c/3) whenever safely achievable.^3^

Intra-arterial thrombolysis (IAT), by directly delivering fibrinolytics at the site, or close to the site, of the residual occlusion, offers a potential adjuvant strategy for the treatment of remaining vessel occlusion, which may improve macro- and potentially micro-vascular perfusion of the hypoperfused brain tissue.^4–8^ In particular, Tenecteplase (TNK), with its high fibrin specificity, resistance to plasminogen activator inhibitor-1, and longer half-life, enables single-bolus administration, making it potentially well suited for the intra-arterial route.^9–11^

Although early observational studies suggest that IAT may improve outcomes without increasing bleeding risk,^4,12,13^ results from recently published or presented RCTs have been mixed—likely due to differences in patient populations as well as the administration of different drugs and dosages.^14–20^ A pooled study-level meta-analysis of these trials indicated a potential benefit in the rate of excellent clinical outcomes, defined as reaching a modified Rankin scale (mRS) score of 0 or 1 at 90 days,^21,22^ but evidence remains limited for patients pretreated with intravenous fibrinolytics, posterior circulation strokes, patients with incomplete reperfusion, the possible effect on microvascular status, and also considering that most trials did not use or only partially use a local injection regimen.

The Safety and Efficacy of Intra-Arterial Tenecteplase for Non-Complete Reperfusion of Intracranial Occlusions (TECNO) trial will assess the efficacy and safety of distally administered intra-arterial TNK in patients with suboptimal reperfusion (eTICI 2a–2c) and persistent, mechanically unamenable occlusions, compared to no further interventional treatment (standard of care).

## Methods

### Study design

TECNO is a multicenter, prospective, randomized (1:1), surrogate-endpoint, open-label, controlled trial with blinded endpoint assessment (PROBE design). In the trial two imaging-based co-primary outcomes are used. The investigational arm involves intra-arterial administration of TNK following incomplete mechanical reperfusion, while patients in the control arm receive no further intra-arterial treatment after MT. In the control group, there is no placebo treatment (see Supplemental Information I for rationale). The trial is registered with ClinicalTrials.gov (NCT05499832) and SNCTP (SNCTP000004994) and is currently being conducted across 35 (planned 42) tertiary care, comprehensive stroke centers offering MT in five (planned 7) countries. Patient enrollment began in March 2023. The current protocol version in Switzerland is V3.1 (22 February 2024) and V3.4 (5 November 2024) in the European Union.

### Participant population

TECNO will include 156 people who underwent MT for an intracranial vessel occlusion (intracranial internal carotid artery, first, second, and third segment of the middle cerebral artery and first and second segment of the anterior cerebral artery and posterior cerebral artery) and show one or multiple residual, non-mechanically amendable occlusions which persist or are newly encountered after MT is performed. All in and exclusion criteria are listed in Table 1.

**Table 1.** In and exclusion criteria of the TECNO trial.

**Inclusion criteria**
Informed consent obtained
Age ⩾ 18 years
Clinical signs consistent with acute ischemic stroke
Initial vessel occlusion in intracranial ICA, M1, M2, M3, A1, A2, P1, or P2
Underwent endovascular stroke treatment
Onset to randomization < 705 min (11 h 45 min) from symptom onset or last seen well
Incomplete reperfusion without mechanically amendable occlusion, as defined by the interventionalist: ICA/M1: TICI 2b/2c (50%–99%) A1/A2, P1/P2, M2/M3: TICI 2a/2b/2c (1%–99%) or emboli to new territory
ASPECTS ⩾ 5 on non-contrast CT or DWI-ASPECTS ⩾ 4 (ASPECTS region involvement ⩾ 20%)^a^
**Exclusion criteria**
Acute intracranial hemorrhage
Contraindication to MRI (e.g., pacemaker)
Severe bleeding within past 6 months
Major surgery within past 2 months with high bleeding risk
Vitamin K antagonist use with INR > 1.7
Platelet count < 50,000/μL
Uncontrolled hypertension (SBP > 185 mmHg or DBP > 110 mmHg despite treatment)
Active dyspeptic ulcer
Known arterial aneurysm
Known neoplasm with bleeding risk
Severe liver fibrosis or portal hypertension
Acute pericarditis
Acute endocarditis
Acute pancreatitis
Known allergy to TNK, gentamicin, or excipients (Polysorbatum 20, L-Argininum, Acidum phosphoricum)
Renal failure (serum creatinine > 3.0 mg/dL, GFR < 30 mL/min, or dialysis-dependent)
Radiologically confirmed mass effect or intracranial tumor (except small meningioma)
Radiologically confirmed cerebral vasculitis
Calcified thrombi
Pregnancy or lactation

ICA: internal carotid artery; TICI: thrombolysis in cerebral infarction; ASPECTS: Alberta Stroke Program Early CT Score; SBP: systolic blood pressure; DBP: diastolic blood pressure; TNK: tenecteplase.

^a^An ASPECTS region (e.g., M3) will be counted as “infarcted” and is subsequently subtracted from the total score of 10 if in that region (e.g., within the ASPECTS region M3) the DWI lesion extends 20% of the overall volume of this ASPECTS region.

### Randomization and blinding

**Figure 1.**
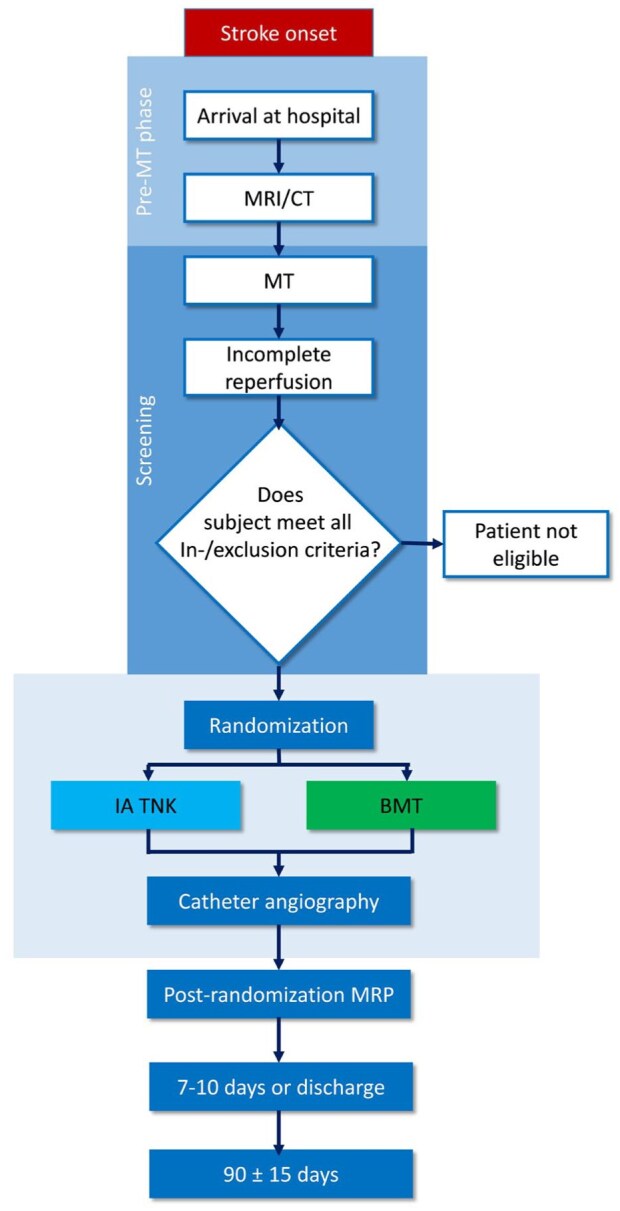
Study flowchart. BMT: best medical treatment; IA: intra-arterial; TNK: tenecteplase; MRP: MRI perfusion; MT: mechanical thrombectomy.

Randomization will be prompted once the potential participant has fulfilled all in- and exclusion criteria, of which final reperfusion grade can only be assessed directly in the angiography suite (see Figure 1 for study flow chart). Randomization is possible up to 11 h and 45 min after symptom-onset or last-seen well (corresponding to an anticipated study drug administration in the interventional arm earlier than 12 h after symptom-onset or last-seen well). The two treatment arms are: (1) IAT with intra-arterial administration of 3 mg TNK and (2) Standard of care without further interventional procedures. Allocation of patients will be done with an online data management system (secuTrial^®^) using probabilistic minimization with a random element of 10%. The allocation of a patient is displayed to the treating physicians in real-time after randomization. Minimization will be done by center, using the following variables: (1) IV tPA or TNK use before MT (yes/no), (2) primary occlusion site before MT (ICA vs M1 vs M2/M3/A1/A2/P1/P2), and (3) single versus multiple occlusions causing incomplete reperfusion (defined as eTICI2a/2b50/2b67/2c reperfusion, depending on the initial occlusion site).

An independent and blinded certified personnel perform assessment of clinical outcomes during clinical visits or a telephone interview. A blinded, independent central imaging core lab (eppData, Hamburg, Germany) evaluates clinical imaging data, including the two primary imaging-based outcomes, in accordance with a prespecified corelab charter and a dedicated training period of the readers with non-trial data. The treating interventional team is not blinded to group allocation and unblinding can be performed for other members of the treating team in case of complications (e.g., intracranial hemorrhage).

### Treatment

The experimental intervention is intra-arterial administration of 3 mg of TNK (Metalyse, Boehringer–Ingelheim) using a microcatheter for distal intra-arterial injection. The rational for not using a weight-adjusted dose was the general low dose and the intra-arterial application route. TNK will be sourced from the market (Metalyse^®^, 50 mg/10 mL) or is directly provided to the centers by Boehringer–Ingelheim within the framework of this trial (40 mg/8 mL). The TNK solution (1000 U/mL, 5 mg/mL) will be prepared according to the manufacture’s instructions. Of this TNK solution, 0.6 mL (equals 3 mg) will be used for microcatheter injection, which is performed over 1 minute. Any CE approved microcather can be used and the choice of the microcatheter is up to the treating interventionalist. After IA TNK is administrated, the dead space volume of the microcatheter is flushed using an injection speed of 1 mL/min so that the TNK will completely enter the distal cerebral artery blood stream. Injection site should be as distal as possible in relation to the residual occlusion. In case of multiple remaining occlusions, the most distal bifurcation covering both territories with the distal vessel occlusions will be used as injection site (Supplemental Figure 1). The full dose of 3 mg is to be administered with the exception of clinical signs or radiological signs of hemorrhages during administration, in which case diagnosis of such a complication is of priority and administration of the drug may be withheld. Micro-catheterization should be finished within 15 min after randomization.

In both arms, patients are treated according to international guidelines.^3^ The use of antiplatelet medication for the treatment (percutaneous transluminal angioplasty and/or stenting) of extracranial stenosing atherosclerotic disease of the internal carotid artery or the vertebral artery was permitted.

### Primary outcomes

**Figure 2.**
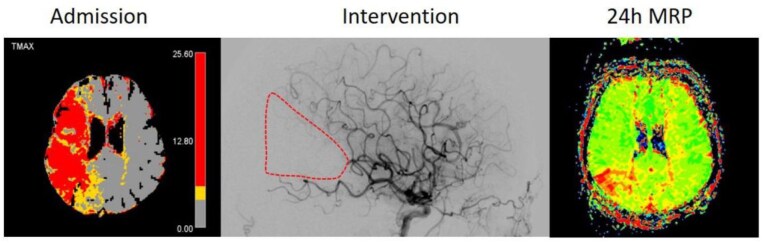
Late reperfusion assessment. Evaluation of 24 h MRI perfusion using Time to Peak (TTP) maps. Patient with initial M1 occlusion an eTICI2b67 reperfusion after MT. Follow-up MRI at 24 h reveals a wedge-shaped TTP deficit, meaning that complete late reperfusion has not occurred. For this patient the co-primary endpoint of late reperfusion was rated as negative/no late reperfusion.

The co-primary outcomes are imaging-based, defined as early and late reperfusion. Early reperfusion is evaluated using full brain coverage anterior-posterior and lateral projection diagnostic angiography runs performed 25 min after randomization using standard injection into the guiding catheter placed in the internal carotid artery (see Supplemental Information II for rationale explaining why the control run is anchored to the time point of randomization). The residual hypoperfused territory due to distal occlusions causing incomplete reperfusion after MT will be defined as new target territory (nTT). This can be within the territory for which MT was envisaged, an embolus to a new territory or both. For more than one residual occlusion the residual hypoperfused territories will be combined to one nTT. This definition is independent from the initial occlusion site. Reperfusion of the nTT at 25 min after randomization will be evaluated applying the most recent modification of the eTICI scale.^1^ Early reperfusion will be a dichotomous outcome of patients achieving eTICI2a-3 versus eTICI 0-1 with respect to the nTT. Late reperfusion will be assessed on 24 h ± 6 h follow-up magnetic resonance perfusion imaging (MRP) using a gradient echo perfusion sequence with administration of gadolinium-based contrast agent. A standard gradient-echo perfusion sequence as per site standard can be used. For post-processing the centers standard perfusion software and a centralized post-processing using Veocore (VEObrain GmbH, Freiburg, Germany) is used. If MRP is not available or clinically not feasible, a computed tomography perfusion (CTP) can serve as a substitute. Complete reperfusion is defined as absence of a focal wedge-shaped TTP and TMAX delay suggestive of a residual occlusion within the nTT as defined above. Complete reperfusion is a dichotomous outcome (present or absent, for an example of the absence of complete reperfusion at 24 h ± 6 h see Figure 2).^23–25^

### Secondary outcomes

The secondary outcomes are clinical and imaging based. These include: (1.1) All-cause mortality at 90 days, (1.2) Degree of disability at 90 ± 15 days as assessed by the mRS (shift analysis), (1.3) Change in NIHSS at 24 h ± 6 h and at 90 ± 15 days, (1.4) Quality of life as assessed by the EuroQol 5D-3L at 90 ± 15 days. Imaging-based secondary outcomes are: (2.1) eTICI reperfusion grade of the new target territory, (2.2) eTICI reperfusion grade of the initial target occlusion, (2.3) infarct growth, defined as difference between DWI positive volume on admission MRI/or CTP positive core on admission CTP and follow-up DWI on 24 h ± 6 h follow-up (difference in mL). An overview regarding assessment time points and clinical visits can be found in Supplemental Table 1.

### Safety outcomes

The safety outcomes are: (1) adverse events of special interest (see Supplemental Table 2), (2) serious adverse events within 90 days, (3a) any intracranial hemorrhage up to 24 h ± 6 h visit, (3b) symptomatic intracranial hemorrhage according to the Heidelberg bleeding classification and as assessed centrally by an independent clinical event committee, (3c) asymptomatic intracranial hemorrhage, and (3d) severe and moderate systemic bleedings (according to the GUSTO criteria).^26^

### Hypothesis and sample size calculation

The primary hypothesis is that patients in the experimental arm have superior early or late reperfusion as compared to patients receiving standard of care (control arm). The study uses two primary endpoints and it is sufficient to demonstrate an effect for one of them. A Bonferroni correction is used to account for type-I error inflation due to multiplicity (alpha set to 0.025).

The sample size is based on expecting an early reperfusion of the residual occlusion in 40% of the IA TNK group (experimental group) and 15% in the control group 25 min after randomization as well as expecting late reperfusion on magnetic resonance perfusion imaging at 24 h in 80% of the IA treatment arm and in 50% of the control arm. Based on these assumptions, a dropout rate of 3.0% and a Chi-squared test with an alpha of 0.025 to adjust for multiplicity, the study will have a power of 89.2% (lowest power for one of the two endpoints).

### Data safety monitoring board and interim analysis

An independent DSMB is monitoring the trial. There will be a safety interim analysis at 40 and 80 patients and it is prespecified that the DSMB will non-bindingly recommend to stop the trial if the probability that the prevalence of symptomatic intracranial hemorrhage is >12% in the experimental arm exceeds 80%. For the assessment of futility, there will be one interim assessment for after 80 participants were randomized .The recommendation of the DSMB to the Sponsor will be to stop the trial for futility, if the conditional power to detect superiority in one of the primary outcomes is <20%.

### Statistical analysis

We will calculate the proportion of patients meeting the criteria of early and late reperfusion in both groups with a corresponding 95% Wilson score confidence interval (CI). For the comparison between the two groups, we will calculate a Mantel–Haenszel risk difference (IA TNK-BMT) with a two-sided 97.5% CI according to the procedure described by Klingenberg,^6^ a Mantel–Haenszel risk ratio with a two-sided 97.5% CI according to Greenland and Robins and a *p*-value from a Cochrane–Mantel–Haenszel test, all stratified for the minimization factors used in randomization. If the lower limit of the 97.5% CIs of the risk difference of one of the primary outcomes lies above 0%, we will claim superiority of the experimental arm. The primary outcome will be analyzed for effect modification by the minimization factors used for randomization and the time from stroke symptom onset to randomization.

### Study organization and funding

The TECNO study is an academic, investigator-initiated clinical trial sponsored by the University Hospital Bern (Inselspital). The trial is supported by grants from the Swiss National Science Foundation, the Dutch Heart Foundation, and Boehringer–Ingelheim (Ingelheim, Germany). None of the funding organizations are involved in the design, conduct, or interpretation of the trial. Trial management is carried out by the Neuro Clinical Trial Unit at the Department of Neurology, University Hospital Bern, Switzerland. The database management, central data monitoring, and statistical analyses are conducted by the Department of Clinical Research at the University of Bern, Switzerland.

### Ethical approval

Ethical approval for the study was first obtained from the Cantonal Ethics Commission (KEK) in Bern, Switzerland (ID 2022-00388), and subsequently from all relevant local ethics committees and, where applicable, from national lead ethics committees and competent regulatory authorities at each participating site.

### Trial status

As of 10 June 2025, 74 patients have been enrolled in the trial. Baseline characteristics of the first 40 randomized patients (included in the first interim analysis) are presented in Supplemental Table 3, while the accrual chart is depicted in Supplemental Figure 2.

## Discussion

The use of IAT during or after thrombectomy has gained increasing attention. While initial observational reports did not find an increased risk of bleeding in patients undergoing thrombectomy who were additionally treated with IAT, indication bias and other limitations related to the observational design limited the translation of these reassuring results into clinical routine.^4,5^ As of now, seven RCTs evaluating IAT in the setting of MT have been published or were recently presented: The Chemical Optimization of Cerebral Embolectomy trial (CHOICE, NCT03876119),^14^ The Intra-arterial Tenecteplase Following Endovascular Reperfusion For Large-Vessel Occlusion Acute Ischemic Stroke trial (POST-TNK, ChiCTR2200064809),^16^ The Adjunctive Intra-arterial Urokinase after Successful Endovascular Thrombectomy in Patients with Large Vessel Occlusion Stroke trial (POST-UK, ChiCTR2200065617),^15^ The Intra-arterial Tenecteplase Following Endovascular Therapy in Patients with Acute Posterior Circulation Arterial Occlusion Trial (ATTENTION-IA, NCT05684172),^19^ The Phase Ib/IIa Safety and Efficacy of Adjunctive Intra-arterial Tenecteplase following Successful Thrombectomy in Patients with Large Vessel Occlusion (DATE, ChiCTR2400080624),^20^ The Intra-arterial Tenecteplase after Successful Endovascular Therapy trial (ANGEL-TNK, NCT05624190),^18^ and the Intra-arterial Alteplase for Acute Ischaemic Stroke after Mechanical Thrombectomy trial (PEARL, NCT05624190).^17^

In four of the above-mentioned trials, the efficacy and safety of intra-arterial TNK has been evaluated, using different dosages and modes of intra-arterial injection.^16,18–20^ In TECNO, a non-weight adjusted dose of 3 mg is used, which was deduced from intra-arterial alteplase dosages found in observational reports of patients treated with MT.^13^ In the phase 1/2 DATE trial, which is the only available dose finding trial for intra-arterial TNK in the setting of MT, the authors concluded that low dose (0.03125 mg/kg) and medium dose (0.0625 mg/kg) TNK showed adequate safety to be tested in larger trials (presented at the International Stroke Conference, ISC, February 5–7, 2025, Los Angeles, CA, USA). Hence, for stroke patients of 60, 65, 70, 75, 80, 85, and 90 kg body weight, the fixed dose used in TECNO lies well within the range of these two suggested doses (Supplemental Table 4). However, the dose is certainly lower than what has been applied in the ANGEL-TNK trial (0.125 mg/kg), which did not show evidence of an increased bleeding risk (5.6% vs 6.2%, for the IA TNK and control group, respectively). Here, the doses applied in TECNO are expected to be twice to four times lower as compared to ANGEL-TNK, depending on the exact weight of the patient.

Out of the six published or presented phase II/III trials, three trials (ANGEL-TNK,^18^ PEARL,^17^ CHOICE^14^) found that IAT significantly increased the rate of mRS 0–1 (presented at the ISC 2025), while the other three trials reported neutral results.^15,16,19^ A study-level meta-analysis of the above-mentioned trials, incorporating the results of the phase 1/2 trial DATE, concluded that IAT after successful MT is associated with higher likelihood of mRS 0–1, without significant safety concerns.^21^ However, only 23% of the patients included had a TICI2b reperfusion result and less than 10% were treated with intravenous tPA or TNK before MT. Furthermore, only around half of the patients were included in trials randomizing to intra-arterial TNK.^21^ Because of that, and due to the fact that the pathophysiological mode of action of IAT in patients with successful but incomplete reperfusion remains to be determined, further evidence seems warranted. In line with these conflicting results, a recent Editorial including a study-level meta-analysis of three of the above mentioned trials concluded that currently ongoing trials including Caucasian participants are awaited to provide more definitive answers and to settle discussions related to generalizability of the reported findings.^27^

The TECNO trial, together with other ongoing randomized controlled trials (see Supplemental Table 5 for an overview), will add to the growing body of evidence regarding the efficacy and safety of intra-arterial TNK in the setting of MT. In particular, the study design helps to estimates its efficacy in a patient population with incomplete reperfusion and can shed light into its likely mode of action as well as the value of a distal local injection regimen.

## Supplementary Material

ds-eso_23969873251381974
