# Supplementary material for: Safety and efficacy of intra-arterial tenecteplase for non-complete reperfusion of intracranial occlusions: Methodology of a randomized, controlled, multicenter study
Source: Eur Stroke J. 2026 Jan 1;11(1):23969873251381974. doi: 10.1093/esj/23969873251381974 (PMC12866206; doi:10.1093/esj/23969873251381974)
Supplement: ds-eso_23969873251381974 [file ds-eso_23969873251381974.zip › 23969873251381974.suppl]

Supplemental Material


## Supplemental Material

Please find the following supplemental material visualised and available to download via Figshare in the display box below. Where there are more than one item, you can scroll through each tab to see each separate item.

Please note all supplemental material carries the same license as the article it is here associated with

- Supplemental Material
