# Supplementary material for: Safety and efficacy of intra-arterial tenecteplase for non-complete reperfusion of intracranial occlusions: Methodology of a randomized, controlled, multicenter study
Source: Eur Stroke J. 2026 Jan 1;11(1):23969873251381974. doi: 10.1093/esj/23969873251381974 (PMC12866206; doi:10.1093/esj/23969873251381974)
Supplement: ds-eso_23969873251381974 [file ds-eso_23969873251381974.zip › sj-docx-1-eso-10.1177_23969873251381974.docx]

**Safety and Efficacy of Intra-Arterial Tenecteplase for Non-Complete Reperfusion of Intracranial Occlusions: Methodology of a randomized, controlled, multicenter study**

**Supplementary Material**

**Supplementary Information**

**Supplementary Information I: Rational for not using a placebo**

If residual occlusions after incomplete mechanical thrombectomy are not amendable to further mechanical bail-out strategies there is currently no evidence based treatment option for these patients. Hence, patients in the control group are offered best medical treatment, as per current standard of care. One alternative consideration for a control group is conceivable: The control group may consist of IA infusion of placebo, which (1) allows for adequate blinding of the operator and (2) will allow for a more adequate comparison of the actual drug effect as opposed to mechanical effects due to infusion of small amount of liquids into occluded arteries. However, catheterization of intracranial vessel and potential blockage of distal vessels in the placebo group is not without risks and does not reflect current standard of care. Thus, using IA placebo infusion in the control groups inevitably comes at the risk of a less favorable outcome in the placebo group than what could have been expected as per current standard of care (i.e. no further treatment/intervention). The choice of the comparator thus is a weighting of blinding and nailing down observed effects to the drug rather than mechanical effects due to general liquid infusion vs. risking complications in patients where standard of care suggest no further treatment. In line with the investigators of the pivotal IA thrombolysis trials, we consider patient safety and estimation of a true effect size compared to current standard of care as more important than the other aforementioned factors, e.g. adequate operator blinding.

**Supplementary Information II: Rationale for anchoring the angiographic control run to the time point of randomization**

As the control group does not receive a placebo infusion, anchoring the follow-up angiogram to the time of randomization provides a standardized and unbiased assessment point for both groups. This approach is critical for controlling for spontaneous reperfusion, which can occur independently of any treatment. As has been recently reported, the Thrombolysis in Cerebral Infarction (TICI) score can change in over 15% of cases within just 10 minutes, a rate that would likely increase with a longer waiting period.^1^ A synchronized 25-minute assessment allows us to isolate the treatment effect from these spontaneous changes. To ensure that we still capture the drug's early pharmacological effects, our protocol mandates that intra-arterial TNK administration (including microcatheter placement and a 1-minute infusion) be completed within 15 minutes of randomization. Consequently, the control angiogram at 25 minutes post-randomization is timed to occur, on average, approximately 10 minutes after the completion of the TNK infusion. If this is sufficient to observe its early impact on reperfusion is not completely clear, but observational data highlighted that some reperfusion after intra-arterial thrombolysis may occur early.^2,3,4^ E.g. in ALLY, a diagnostic control run was performed as early as 5 minutes after administration of intra-arterial and reperfusion was observed in some cases.^4^ While a longer observation period post-infusion was considered, it was deemed incompatible with standard interventional workflows and could potentially increase procedural risks. Therefore, the 25-minute post-randomization endpoint represents a necessary and pragmatic compromise, balancing the capture of early treatment effects with the need for a robust control comparison and procedural safety. To capture later reperfusion a co-primary endpoint using 24h perfusion imaging was established.

**References**

1. Midtlien JP, Ashraf O, Hui FK, Tsappidi S, Zhang YJ, Forster AA, Chang E, Wiater AH, Starke RM, Abdelsalam A, et al. One in six patients exhibit changes in reperfusion on 10-minute repeat cerebral angiography during mechanical thrombectomy for stroke. J. Neurointerv. Surg. [Internet]. 2025;Available from: http://dx.doi.org/10.1136/jnis-2024-022448

2. Kaesmacher J, Abdullayev N, Maamari B, Dobrocky T, Vynckier J, Piechowiak EI, Pop R, Behme D, Sporns PB, Styczen H, et al. Safety and angiographic efficacy of intra-arterial fibrinolytics as adjunct to mechanical thrombectomy: Results from the INFINITY registry. J. Stroke. 2021;23:91–102.

3. Kaesmacher J, Bellwald S, Dobrocky T, Meinel TR, Piechowiak EI, Goeldlin M, Kurmann CC, Heldner MR, Jung S, Mordasini P, et al. Safety and efficacy of intra-arterial urokinase after failed, unsuccessful, or incomplete mechanical thrombectomy in anterior circulation large-vessel occlusion stroke. JAMA Neurol. 2020;77:318–326.

4. Zaidi SF, Castonguay AC, Zaidat OO, Jadhav AP, Sheth SA, Haussen DC, Nguyen TN, Burgess RE, Alhajala HS, Gharaibeh K, et al. Safety of Adjunctive Intraarterial Tenecteplase Following Mechanical Thrombectomy: The ALLY pilot trial. Stroke. 2025;56:355–361.

**Supplementary Information III: Author Disclosures**

JK reports a research grant from Boehringer-Ingelheim (Ingelheim am Rhein, Germany) supporting the IRIS collaboration and the TECNO trial, financial support within a research agreement with Siemens (Forchheim, Germany) related to flat-panel imaging, research support by Le Studium (Orleans, France) for a research fellowship, a research collaboration without financial compensation with Cercare (Copenhagen, Denmark), a research grant from the Bangerter-Rhyner foundation (Bern, Switzerland) supporting a project on individualized decision making on intravenous thrombolysis, a research grant from the Swiss Heart Foundation (Bern, Switzerland) supporting the Prediction of Delayed Reperfusion using Flatpanel imaging and research grants from the Swiss National Science Foundation (Bern, Switzerland) supporting the TECNO and DO-IT trial as well as a research grant provided by the Horten Foundation (Zurich, Switzerland) to support the DO-IT trial; all fees are paid to the institution. UF reported research support of the Swiss National Science Foundation and the Swiss Heart Foundation. PI of the ELAN trial, Co-PI of the DISTAL, TECNO, SWIFT DIRECT, SWITCH, ELAPSE and ICARUS trial. Research grants from Medtronic (BEYOND SWIFT, SWIFT DIRECT) and from Stryker, Rapid medical, Penumbra, Medtronic and Phenox (DISTAL), Boehringer Ingelheim (TECNO). Support of the Horton Foundation for the DO IT trial. Consultancies for Medtronic (fees paid to institution). Participation in an advisory board for AstraZeneca (former Alexion/Portola), Bayer, Boehringer Ingelheim, Biogen, AbbVie, Siemens (fees paid to institution). Member of a clinical event committee (CEC) of the COATING study (Phenox). Member of the data and safety monitoring committee (DSMB) of the TITAN, LATE_MT, IN EXTREMIS and RapidPulse trials. President of the Swiss Neurological Society and president-elect of the European Stroke Organisation. EIP reports research grants of the Swiss National Science Foundation. AM reports financial support from the Swiss National Science Foundation, Department for Learning and Teaching of Inselspital Bern and Swiss Heart Foundation. All fees are paid to the institution. GT reports honoraria as consultant or lecturer from Acandis, Astra Zeneca, Bayer, BristolMyersSquibb/Pfizer, Boehringer Ingelheim, Daiichi Sankoy, and Lilly. MNP received research grants from the Swiss National Science Foundation (SNF) and Bangerter-Rhyner Stiftung, unrestricted grant support from Medtronic Inc., Rapid Medical Inc., Penumbra Inc., Siemens Healthineers AG, Stryker Neurovascular Inc., Phenox GmbH (paid to institution) and speaker fees from Stryker Neurovascular Inc., Medtronic Inc., Penumbra Inc., Acandis GmbH, Phenox GmbH, Rapid Medical Inc., Siemens Healthineers AG (paid to institution). He is the Sponsor-PI of the DISTAL, SPINNERS and ICARUS Trials. HK reports compensation as speaker from Asklepios Kliniken, travel funding from Penumbra, Inc., an ownership stake in Eppdata GmbH and compensation from Eppdata GmbH for consultant services. All other authors report not disclosures.

**Supplementary Information IV - Consent Process in different countries**

Consent procedures in Switzerland, Belgium, the Netherlands and Portugal for patients in emergency situations, according to local ethic policy:

|  | **Requirement  for trial inclusion ^1)^** | **Post-hoc consent  during initial hospitalisation or day of discharge ^2)^** | | **Post-hoc consent visit 4**  **(day 90 ± 15 days) ^3)^** | |
| --- | --- | --- | --- | --- | --- |
| **Country** | Independent  physician | Patient | Next of kin/  LAR | Patient | Next of kin/  LAR |
| Switzerland | 🗸 | 🗸* | 🗸* | (🗸) | (🗸) |
| Belgium | Deferred consent | 🗸* | 🗸* | (🗸) | (🗸) |
| the Netherlands | Deferred consent | 🗸* | 🗸* | (🗸) | (🗸) |
| Portugal | Deferred Consent | 🗸* | 🗸* | (🗸) | (🗸) |

1) 🗸 Mandatory for trial inclusion

2) 🗸* Either or, minimal requirement for post-hoc consent

3) (🗸) Either or, minimal requirement for post-hoc consent if not obtained during initial

hospitalisation

1. When a patient is presenting to the emergency room with an acute ischaemic stroke being a possible candidate for the trial by judgement of the responsible trial physician, the patient can be enrolled into the trial without prior consent of the patient/legal representative as per deferred consent procedure in accordance with CTR EU 536/2014 Article 35 Clinical trials in Emergency Situations.
2. Post hoc consent during initial hospitalisation of the patient is mandatory and has to be asked for as soon as the patient is capable to give consent after trial inclusion. This means either during the initial hospitalization or at visit 3/day of discharge. If the patient is not capable to consent before his/her discharge, the informed consent of the patient’s next of kin/LAR must be obtained during this period (if necessary, the center must explore the possibility of obtaining remote consent via a secure link from the next of kin/LAR). If the patient is unable to write, consent may be given and recorded through appropriate alternative means in the presence of at least one impartial witness. In that case, the witness shall sign and date the informed consent document.
3. Post hoc consent will be asked at visit 4.
   1. All attempts under 2) were carried out but were not successful.
   2. If the patient does not regain the capability to consent before his/her discharge, his/her consent must be asked at the clinical visit 4, even if the next of kin/LAR gave his/her consent before.
   3. If the patient is not capable to consent at visit 4 and no consent from the next of kin/LAR could be obtained up to this period, post hoc consent of a next of kin /LAR is mandatory.
   4. If the patient is not capable to consent at visit 4, and only then, the informed consent of the patient’s next of kin/LAR is considered to be sufficient, but mandatory.

If the subject or, where applicable, his or her legally designated representative does not give consent, he or she shall be informed of the right to object to the use of data obtained from the clinical trial in accordance to Article 35 point 3 of the CTR.

Consent procedures in Germany, Finland and Austria for patients in emergency situations, according to local ethic policy:

|  | **Requirement  for trial inclusion ^1)^** | **Post-hoc consent  during initial hospitalisation ^2)^** | | **Post-hoc consent  day of discharge and visit 4 ^3)^** | | **Post-hoc consent visit 4**  **(day 90 ± 15 days) ^4)^** | |
| --- | --- | --- | --- | --- | --- | --- | --- |
| **Country** | Independent  physician | Patient | Next of kin/  LAR | Patient | Next of kin/  LAR | Patient | Next of kin/  LAR |
| Germany | 🗸 | 🗸* | 🗸* | 🗸** | 🗸** | (🗸) | (🗸) |
| Finland | No consent needed | 🗸* | 🗸* | 🗸** | 🗸** | (🗸) | (🗸) |
| Austria | No consent needed | 🗸* | 🗸* | 🗸** | 🗸** | (🗸) | (🗸) |

1) 🗸 No consent needed for trial inclusion

2) 🗸* Either or, minimal requirement for post-hoc consent

3) 🗸** Either or, minimal requirement for post-hoc consent if not obtained during initial hospitalisation/day of discharge

4) (🗸) Either or, minimal requirement for post-hoc consent if not obtained during initial hospitalisation

1. When a patient is presenting to the emergency room with an acute ischaemic stroke being a possible candidate for the trial by judgement of the responsible trial physician, the patient can be enrolled into the trial.
2. Post hoc consent during initial hospitalisation of the patient is mandatory and has to be asked for as soon as the patient is capable to give consent after trial inclusion. This means either during the initial hospitalization or at visit 3/day of discharge. If the patient is not capable to consent before his/her discharge, the informed consent of the patient’s next of kin/LAR must be asked during this period (if necessary, the sponsor must explore the possibility of obtaining remote consent via a secure link from the next of kin/LAR). If the patient is unable to write, consent may be given and recorded through appropriate alternative means in the presence of at least one impartial witness. In that case, the witness shall sign and date the informed consent document.
3. In case the patient was not capable to consent during hospitalization at visit 3/day of discharge and no consent from the next of kin/LAR could be obtained up to this period the following attempts need to be done at least once per month between particpant’s discharge from hospital/visit 3 and visit 4
   1. Contact the patient and obtain consent if his/her condition has changed so he/she is capable to give consent.
   2. If the patient is not capable to give consent, contact and obtain consent from the legally designated representative/relative or the patient during the time period between participant´s discharge from hospital/visit 3 and visit 4.

For a) and b): All efforts should be done to obtain consent as fast as possible and all available options for contact should be used to obtain this goal. If necessary, the sponsor must explore the possibility of obtaining remote consent via a secure link from the patient or/and next of kin/LAR.

1. Post hoc consent at visit 4 is mandatory in case of an absent post hoc consent during the initial hospitalisation and the time between day of discharge and visit 4.
   1. All attempts under 3) were carried out but were not successful.
   2. If the patient does not regain the capability to consent before his/her discharge, his/her consent must be asked at the clinical visit 4, even if the next of kin/LAR gave his/her consent before.
   3. If the patient is not capable to consent at visit 4 and no consent from the next of kin/LAR could be obtained up to this period, post hoc consent of a next of kin /LAR is mandatory.
   4. If the patient is not capable to consent at visit 4, and only then, the informed consent of the patient’s next of kin/LAR is considered to be sufficient, but mandatory.

If the subject or, where applicable, his or her legally designated representative does not give consent, he or she shall be informed of the right to object to the use of data obtained from the clinical trial in accordance to Article 35 point 3 of the CTR.

**Supplementary Figures**

**Supplementary Figure 1** – Schematic drawing of injection site in case of multiple residual occlusions


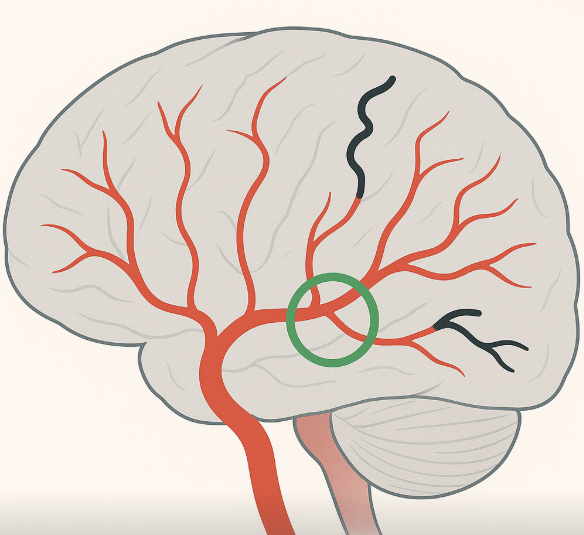


**Supplementary Figure 2** – Accrual Chart
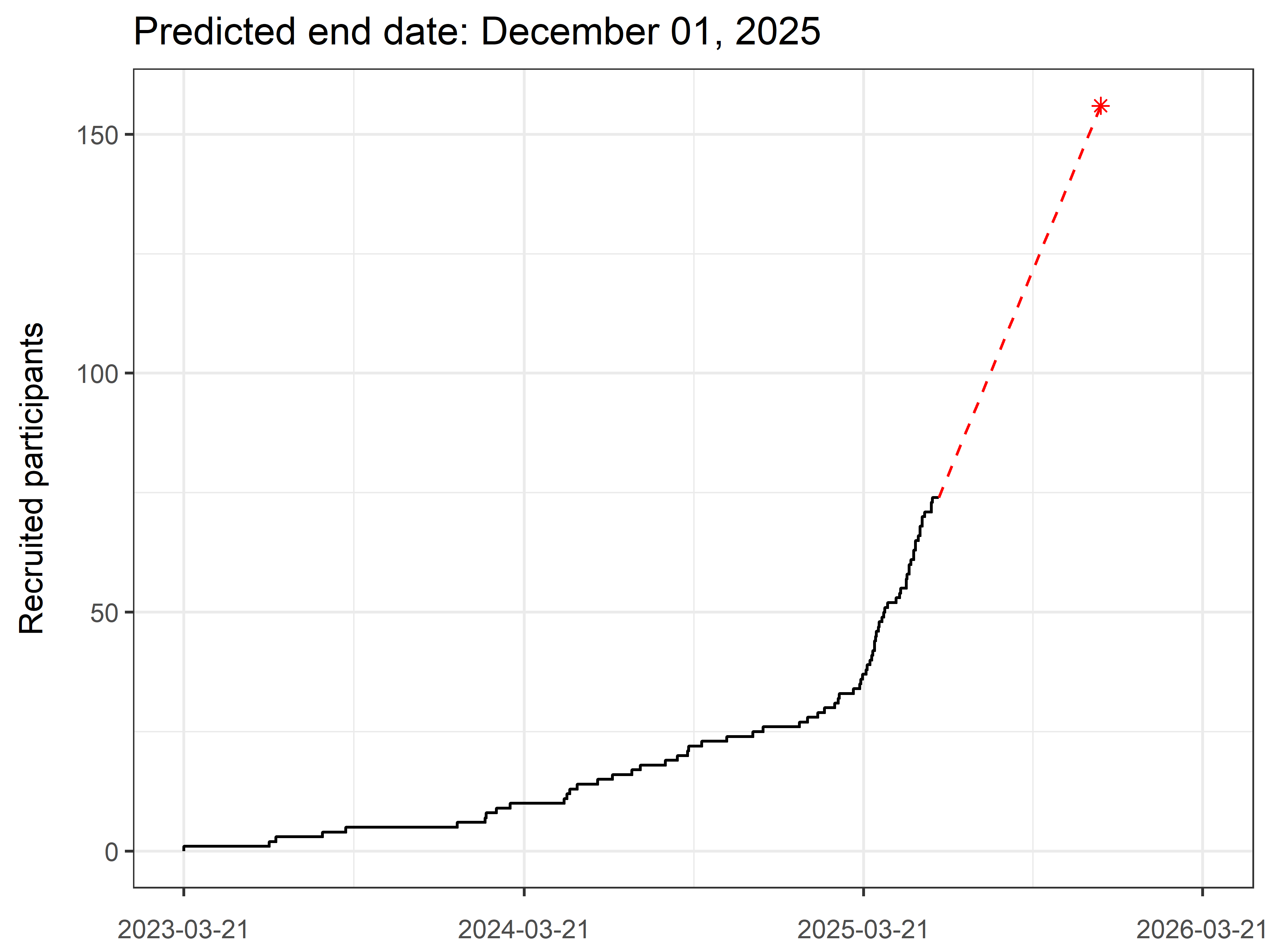


**Supplementary Tables**

**Supplementary Table 1** – Overview of trial visits and procedures

| **Schedule of Assessments** | | | | | | |
| --- | --- | --- | --- | --- | --- | --- |
| **Visits** | | **0** | **1** | **2** | **3** | **4** |
|  |  | **Screening and Randomisation** | **Baseline** | **Post-Randomisation** | **7-10 Days or Discharge** | **90 Days** |
| **Assessment** | Time Window | Day 0 | Day 0 | 24 ± 6 hours | Day 7-10  (if discharge is earlier, at discharge) | Day 90  ± 15 days |
|  | Method | Clinical visit  +  Standard of care intervention | Pharmacological  Intervention | Clinical visit  +  Imaging | Clinical visit | Clinical visit or telephone interview^7^ |
| Eligibility criteria | | x |  |  |  |  |
| Informed Consent | | x |  |  | x^1^ | x^1^ |
| Medical History ^2^ | | x |  |  |  |  |
| Pregnancy Test ^2^ | | x |  |  |  |  |
| Blood Labs ^2^ | | x |  |  |  |  |
| NIHSS ^2^ | | x |  | x^3^ | x^3^ | x^3^ |
| Pre-stroke mRS ^2, 4^ | | x |  |  |  |  |
| Randomisation | | x |  |  |  |  |
| MRI or CT ^2^ | | x |  |  |  |  |
| MRI, including MRP ^5^ | |  |  | x^5^ |  |  |
| ASPECTS ^2, 6^ | | x |  | x |  |  |
| Catheter Angiography ^2^ | | x | x |  |  |  |
| Mechanical thrombectomy | | x |  |  |  |  |
| IA TNK | |  | x^8^ |  |  |  |
| Concomitant Medications ^2^ | | x | x | x | x | x |
| Concomitant Procedure ^2^ | | x | x | x | x | x |
| Serious Adverse Events | | x | x | x | x | x |
| Adverse Events of Interest | | x | x | x | x | x |
| Resource Utilization | |  |  |  | x | x |
| mRS^3^ | |  |  |  | x^3^ | x^3^ |
| EuroQol 5D-3L^3^ | |  |  |  |  | x^3^ |

1. Post-hoc consent of patient (informed consent was given by patient’s next of kin/LAR and/or independent).
2. Routine examination for acute ischemic stroke patients (intravenous blood sampling), depending on the participating hospital, but usually including including blood count, coagulation status, HbA1c, serum sodium, calcium, potassium, glucose and creatinine.
3. Assessment to be performed by an independent evaluator blinded to treatment assignment.
4. Pre-stroke mRS is completed by obtaining verification from an individual aware of the subject’s functional status prior to stroke (e.g. family member, friend, etc.)
5. MR perfusion using gradient-echo sequences and administration of intravenous Gd-contrast agents according to manufacturers’ instructions.
6. ASPECTS scoring has to be performed by a trained physician. The optional use of additional validated software e.g. RAPID is allowed.
7. Clinical visit whenever possible, otherwise telephone interview.
8. If randomized to the interventional arm of the trial.

NIHSS, National Institute of Health Score Scale; mRS, modified Rankin Scale; MRI, Magnet Resonance Imaging; MRP, magnetic resonance perfusion imaging; CT, Computed Tomography; ASPECTS, Alberta Stroke Program Early CT Score; IA TNK, intraartrial tenecteplase.

**Supplementary Table 2** – Adverse Events of Special Interest

| **AE of Special Interest** | **MedDRA codes** |
| --- | --- |
| Haemorrhage at puncture site/vascular access site haemorrhage | 10077653 |
| Aneurysma spurium at puncture site/ vascular access site pseudoaneurysm | 10077649 |
| Intracranial vessel perforation | 10068149 |
| Subarachnoid haemorrhage | 10042316 |
| Extracranial vessel perforation |  |
| Intra- or extracranial dissection of the vessel | 10070693 |
| Intracranial Haemorrhage, including intracerebral, subdural, epidural and subarachnoid haemorrhage |  |
| Extracranial haemorrhage |  |
| Hematoma at puncture site/vascular access site haematoma | 10077647 |
| Haemorrhage at puncture site/vascular access site haemorrhage | 10077653 |
| Bleeding to the pericardium |  |
| Gastrointestinal haemorrhage /Hematemesis | 10019418 |
| Retroperitoneal haemorrhage | 10038980 |
| Urogenital bleeding |  |
| Ecchymoses | 10014079 |
| Eye bleeding | 10071804 |
| Hypovolemic shock | 10021140 |

| **Foreseeable (S)AE related to MT** | **MedDRA codes** |
| --- | --- |
| Hematoma at puncture site/vascular access site haematoma | 10077647 |
| Haemorrhage at puncture site/vascular access site haemorrhage | 10077653 |
| Aneurysma spurium at puncture site/ vascular access site pseudoaneurysm | 10077649 |
| Intracranial vessel perforation | 10068149 |
| Subarachnoid haemorrhage | 10042316 |
| Extracranial vessel perforation |  |
| Intra- or extracranial dissection of the vessel | 10070693 |
| Vascular spasm | 10070702 |
| Air embolism | 10001526 |
| Cerebral vascular occlusion | 10076895 |
| Distal embolization including to a previously uninvolved territory |  |
| Thrombus formation following thrombectomy |  |
| Hypersensitivity to antiplatelet/ anticoagulation agents or contrast media | 10020757 |
| Device deformation, collapse, fracture or malfunction |  |
| Neurologic status (Cognitive?) deterioration | 10064098 |
| Ischemic stroke  - New ischemic infarct  - Infarct growth  - reocclusion  - non-recanalisation | 10061256 |
| Puncture site infection | 10063677 |
| Thrombosis | 10043607 |
| **Foreseeable (S)AE related to IV tPA** | **MedDRA codes** |
| Intracranial Haemorrhage, including intracerebral, subdural, epidural and subarachnoid haemorrhage |  |
| Extracranial haemorrhage: |  |
| - Hematoma at puncture site/vascular access site haematoma | 10077647 |
| - Haemorrhage at puncture site/vascular access site haemorrhage | 10077653 |
| - Bleeding to the pericardium |  |
| - Gastrointestinal haemorrhage / Hematemesis | 10019418 |
| - Retroperitoneal haemorrhage | 10038980 |
| - Urogenital bleeding |  |
| - Ecchymoses |  |
| - Eye bleeding | 10071804 |
| - Hypovolemic shock | 10021140 |
| Reocclusion | 10038563 |
| Angioedema | 10002424 |
| Hematemesis | 10019418 |
| Bradycardia | 10006093 |
| Cardiac arrest | 10007515 |
| Myocardial infarction | 10028596 |
| Haemoptysis | 10018964 |
| Anaphylactoid reaction | 10002216 |
| Skin rash | 10040913 |
| Urticarial | 10046757 |
| Bronchospasms | 10006482 |
| Laryngeal edema | 10023838 |
| Arrhythmias | 10003119 |
| Nausea | 10028813 |
| Vomiting | 10047700 |
| Blood pressure reduction |  |
| Increase in body temperature |  |
| Fatty embolisms |  |

| **Foreseeable (S)AE related to acute ischemic stroke** | **MedDRA codes** |
| --- | --- |
| Hypotenstion |  |
| Visual loss |  |
| Multiorgan failure |  |
| Acute kidney failure | 10000821 |
| Seizures | 10039910 |
| Pulmonary Edema | 10037375 |
| Sepsis | 10040047 |
| Pulmonary Infection / Pneumonia | 10035664 |
| Pulmonary embolism | 10037377 |
| Bradycardia | 10006093 |
| Tachycardia | 10043071 |
| Acute myocardial infarction | 10000891 |
| Hemiparesis left and right | 10019466 / 10019467 |
| Aphesia (motor / sensor) | 10002949/10002951 |
| Dysarthria | 10013887 |
| Dysphagia | 10013950 |
| Hemianopia | 10019457 |
| Neurologic neglect syndrome | 10029289 |
| Pneumonia aspiration | 10035669 |
| Aspiration | 10003504 |
| Infections | 10021878 |
| Cognitive deterioration, impaired consciousness | 10009843 |
| Delirium | 10012218 |
| Persistent vegetative state | 10034715 |
| Brain death | 10049054 |
| Death | 10011906 |
| Cerebral Empyema |  |
| Compression cerebri |  |
| Cerebral edema | 10008107 |

**Supplementary Table 3** – Baseline characteristics of the first 40 randomized patients (first interim analysis)

|  | Total (N = 40) |
| --- | --- |
| Age (years) |  |
| mean (sd) | 73 (12) |
| median [lq, uq] | 76 [62, 82] |
| Sex - n (%) |  |
| Male | 18 (45.0%) |
| Female | 22 (55.0%) |
| IVT thrombolysis used before MT - n (%) |  |
| no | 7 (17.5%) |
| yes | 33 (82.5%) |
| Angiographically visible occlusions causing incomplete reperfusions - n (%) |  |
| Single | 27 (67.5%) |
| Multiple | 13 (32.5%) |
| Occlusion location - n (%) |  |
| ICA | 9 (22.5%) |
| M1 | 10 (25.0%) |
| M2, M3, A1, A2, P1, P2 | 21 (52.5%) |
| Weight (kg) |  |
| mean (sd) | 73 (24) |
| median [lq, uq] | 75 [62, 90] |
| Systolic blood pressure (mmHg) |  |
| mean (sd) | 154 (29) |
| median [lq, uq] | 160 [129, 176] |
| Diastolic blood pressure (mmHg) |  |
| mean (sd) | 78 (15) |
| median [lq, uq] | 78 [66, 92] |
| Heart rate (beat/min) |  |
| mean (sd) | 83 (19) |
| median [lq, uq] | 79 [70, 98] |
| NIHSS |  |
| mean (sd) | 14 (6.5) |
| median [lq, uq] | 13 [9.0, 19] |
| missing - n (%) | 1 (2.5%) |
| Pre-stroke mRS - n (%) |  |
| 0 | 28 (70.0%) |
| 1 | 2 (5.0%) |
| 2 | 4 (10.0%) |
| 3 | 6 (15.0%) |
| Stroke onset to randomisation (min) |  |
| mean (sd) | 271 (93) |
| median [lq, uq] | 253 [194, 331] |

sd: standard deviation, lq: lower quartile, uq: upper quartile.

**Supplementary Table 4** - Comparison of weight-adjusted doses supported by the DATE trial, the Angel-TNK trial and the fixed-dose used in the TECNO trial

| Patient weight  (in kg) | Lower Dose TNK (0.0313 mg/kg) according to DATE  (in mg) | Moderate Dose TNK (0.0625 mg/kg) according to DATE  (in mg) | Dose ANGEL-TNK (0.125 mg/kg)  (in mg) | TECNO TNK regimen  (in mg) |
| --- | --- | --- | --- | --- |
| 60 | 1,88 | 3,75 | 7,50 | 3 |
| 65 | 2,03 | 4,06 | 8,13 | 3 |
| 70 | 2,19 | 4,38 | 8,75 | 3 |
| 75 | 2,34 | 4,69 | 9,38 | 3 |
| 80 | 2,50 | 5,00 | 10,00 | 3 |
| 85 | 2,66 | 5,31 | 10,63 | 3 |
| 90 | 2,81 | 5,63 | 11,25 | 3 |

**Supplementary Table 5** - Overview of the Ongoing Trials on Intra-arterial Thrombolytics

| Name | Design | Thrombolytic | Primary outcome | TICI at inclusion | IVT pre-treatment allowed |
| --- | --- | --- | --- | --- | --- |
| CHOICE-II (NCT05797792) | Phase 3 randomized trial, open, blinded end point assessment | Alteplase (infusion of 1.0 mg/ml over 15 minutes) | Microvascular reperfusion (36±24h CTP) | mTICI 2b-3 | Yes |
| EXTEND-AGNES TNK (NCT05892510) | Phase 2b-3, double-blinded, placebo-controlled, randomized trial | Tenecteplase (0.0625 mg/kg, maximum 6.25mg) | Early neurological improve (NIHSS reduction >4) and mRS 0-2 at 90d | Not specified | Yes, only if infused before 30 minutes of anticipated trial drug |
| IA-SUCCESS (N° EU CT 2023-506935-14) | Phase 3 randomized trial, open, blinded end point assessment | Alteplase (0.225 mg/kg with  maximum authorized dose of 20 mg, over 15 minutes) | mRS shift at 90 d | eTICI 2b-3 | Yes |
| INSIST-IT (NCT05657457) | Phase 3, single-blinded, parallel assignment, randomized trial | Tenecteplase (0.2 or 0.25 or 3 mg/min over 20-30 minutes) | mRS 0–3 at 90 d | mTICI 2b-3 | Yes |
| INSIST-TNK (NCT04201964) | Phase 2, single arm, no masking | Tenecteplase (0.2–0.4 mg/min; 30–40 min duration) | Reperfusion (final TICI 2b-3) | mTICI 1-2a | Not specified |
| RESCUE-TNK (NCT05657470) | Phase 2, single-blinded, parallel assignment, randomized trial | Tenecteplase (0.2–0.3 mg/min, for 20–30 min) | Reperfusion (final eTICI 2b67-3) | Not specified | Yes |
| TECNO (NCT05499832) | Phase 2, single-blinded, parallel assignment, randomized trial | Tenecteplase (3mg TNK, 0.6ml volume, over 1 minute) | Early reperfusion (final TICI score) and delayed reperfusion (24h perfusion imaging) | eTICI 2a-2c | Yes |
| ARTERIAL TNK BAO (NCT05580822) | Phase 2, double-blinded, placebo-controlled, randomized trial | Tenecteplase (0.25 or 0.4 mg/min over 15 minutes) | mRS 0–3 at 90 d | eTICI 2b-3 | No |

mRS, Modified Rankin Scale; NIHSS, National Institutes of Health Stroke Scale; TICI: Treatment in Cerebral Infarction; mTICI: modified TICI; eTICI: expanded TIC
